# Degenerative Mitral Stenosis—Diagnostic Challenges and Future Directions

**DOI:** 10.31083/j.rcm2310354

**Published:** 2022-10-18

**Authors:** Abdulaziz Joury, Christopher Puleo, Anjani Golive, Yvonne Gilliland, Gregg S. Pressman, Salima Qamruddin

**Affiliations:** ^1^Department of Cardiology, Ochsner Medical Center, New Orleans, LA 70118, USA; ^2^King Salman Heart Center, King Fahad Medical City, 12231 Riyadh, Saudi Arabia; ^3^The University of Queensland School of Medicine, Ochsner Clinical School, New Orleans, LA 70121, USA; ^4^Einstein Medical Center, Heart and Vascular Institute, Philadelphia, PA 19140, USA

**Keywords:** degenerative mitral stenosis, echocardiography, rheumatic mitral stenosis

## Abstract

Determining the severity of stenosis in degenerative mitral stenosis (DMS) is fraught with challenges. Neither a high trans-mitral gradient nor a small valve area calculation is sufficiently diagnostic for DMS due to variable left atrial and left ventricular compliance in the setting of diastolic dysfunction, and the variable flow seen in patients with chronic kidney disease (i.e., high flow state) and elderly women (low flow state). Three-dimensional measurement of mitral valve area may be underestimated due to shadowing from basal calcium, and mitral valve annulus (MVA) by continuity equation (CEQ) or dimensionless mitral valve index can be erroneous in the presence of significant regurgitation of left-sided valves. The proposed dimensionless mitral stenosis index (DMSI) can be an easy echocardiographic tool to use in daily practice but needs further validation and is limited in the setting of significant regurgitation of left sided valves. Mean trans-mitral gradients >8 mmHg and pulmonary artery pressure >50 mmHg are independent predictors of mortality in those with MVA <1.5 ${\mathrm{cm}}^{2}$ derived by CEQ. In patients who have symptoms that are out of proportion to the degree of stenosis reported, exercise stress testing may help determine the physiologic effects of the stenotic valve. A combination of MVA by CEQ or DMSI and mean transmitral gradient at a given left ventricle stroke volume (flow) should be evaluated in larger studies.

## 1. Introduction

Mitral annular calcification (MAC) is an increasingly common etiology of mitral valve stenosis in the elderly women and is termed Degenerative Mitral Stenosis (DMS). It is caused by an atherosclerotic-like inflammatory process that occurs as a result of the accumulation of oxidized lipids predominantly in the posterior mitral annulus and leaflet and migrating anteriorly as the disease progresses [1]. Progression of the disease can lead to an extension of this calcification process from the mitral annulus onto the base of the mitral valve (MV) leaflets leading to left ventricle (LV) inflow tract obstruction and mitral valve stenosis [1, 2]. Calcium deposits often form a “shelf” that displaces the mitral valve hinge into the LV inlet, thereby decreasing the mitral valve orifice area and causing DMS. Degenerative mitral stenosis incidence is increasing due to aging population with multiple comorbidities such as chronic kidney disease (CKD), calcium and phosphorus dysregulation. It is associated with higher all-cause mortality [3, 4].

DMS is a different prototype of mitral stenosis (MS) when compared to rheumatic mitral stenosis (RMS). In DMS calcification of the annulus and basal MV leaflets leads to tunnel-like stenosis with the greatest narrowing at the base as MAC progresses and no significant change in mitral valve area at the tips compared to the base at each stage of MAC [5] and with no commissural fusion [6, 7]. Also, the annular area at the base and mid inlet appears to be inversely proportional to the rise in mean trans-mitral gradients with no relation to the MV area at the distal tips [8]. This is in contrast with patients with RMS where commissural fusion, leaflet and chordal thickening occurred—while sparing of the annulus—cause the valve to taper toward its free margins such that it assumes a characteristic funnel shape [9, 10]. This change in morphology from base to tip changes the relationship of mitral valve annulus (MVA) to mean gradient as there is less contraction of flow distal to the stenotic orifice in a prolonged tube or tunnel such as that of DMS. This will lead to lower trans-mitral gradients for a given MVA compared to a flat surface orifice such as that of RMS [11]. In addition, mean gradients are dependent on the pressure difference between the left atrium and LV, and reduced LV compliance frequently seen in the elderly patients might result in lower mean gradients and potentially underestimate true stenosis severity in DMS [12].

The above-mentioned differences between the 2 types of MS create diagnostic challenges as the echocardiographic tools that have been validated in prior studies to be used to diagnose the severity of RMS cannot be applied for the accurate diagnosis of DMS. This review tackles some of the current diagnostic challenges to accurately assess the severity of DMS in the light of echocardiographic predictors of poor prognosis.

## 2. Challenges of Echocardiographic Evaluation of DMS

MAC on echocardiography will frequently manifest as an echo-dense, irregular, and shelf-like structure, predominantly of the posterior annulus, though calcification may extend to the anterior annulus, mitral valve leaflets and LV myocardium [13]. Extension to one-third to one-half of the annular circumference is considered moderate, and larger accumulations are considered severe. Anteroposterior extension of >4 mm thickness in the short axis into the LV inlet is considered severe MAC [14]. American College of Cardiology/American Heart Association valvular heart disease guidelines define severe MS as MVA of ≤1.5 ${\mathrm{cm}}^{2}$ which corresponds to >5 mmHg to 10 mmHg at a normal heart rate [15]. In the following text, we discuss various echocardiographic methods of quantifying the severity of DMS.

### 2.1 Two-Dimensional Mitral Valve Area Planimetry 

Planimetry involves direct measurement of MVA by tracing the smallest anatomical orifice area using 2-D or 3-D imaging. MVA by 2-D planimetry is performed in the parasternal short axis view in mid diastole; the measurement plane should be perpendicular to the mitral orifice. Scanning from base to apex of the LV helps identify the true leaflet tips. In DMS and MAC, the calcification of the base and mid-portion of the MV leaflets can cause significant acoustic shadowing which subsequently result in thinner appreciation of leaflets and thus under-estimation of the MVA [16]. Increased echocardiographic gain settings can further exacerbate acoustic shadowing [17].

### 2.2 Pressure Half Time

Pressure half-time (PHT) is the time interval required for the trans-mitral pressure gradient (TMPG) to reduce to half the peak value during diastole. PHT has an inverse relationship with MVA and it is a well-established method for determining MVA in the setting of RMS [18]. PHT of 220 ms has been shown to be equal to MVA = 1 ${\mathrm{cm}}^{2}$ by the Gorlin equation. Estimation of MVA by utilization of PHT is affected by the compliance of left atrium and LV, and estimated MVA can be increased or decreased based on left atrium and LV compliance [17, 18]. Among patients with DMS who frequently multiple comorbidities have such as diabetes mellitus, CKD, and cardiovascular disease, PHT cannot accurately estimate MVA [19, 20, 21]. Similarly, among DMS patients, left atrial compliance can also vary from normal in these patients (increased LV filling pressure, tachycardia, atrial fibrillation), thus PHT is not routinely recommended in patients with DMS [17].

### 2.3 Proximal Iso-Velocity Surface Area (PISA)

The proximal iso-velocity surface area (PISA) method is based on the concepts of fluid convergence and conservation of mass. As a fluid stream converges towards a narrow orifice, flow accelerates and forms multiple hemispheric shells of increasing velocity and decreasing radius. In order for the mass to be conserved, flow at any of these hemispheric shells must equal flow across the orifice. Incorporating the radius of the convergence hemisphere, aliasing velocity, peak mitral inflow velocity and the opening leaflet angle relative to the flow direction, MVA can be calculated. The iso-velocity shell changes throughout the systole so measuring the largest shell, using frame by frame analysis, can be erroneous. Multiple independent manual measurements can introduce significant errors, even when performed by experienced users [22]. In the setting of coexisting mitral regurgitation, color Doppler assessment of the hemispheric convergence of the mitral diastolic flow on the atrial side of the MV can be technically challenging and requires a high level of expertise. The effect of variable heart rate, such as seen in atrial fibrillation (present in 30% of DMS patients) can further limit its accuracy [17, 23].

### 2.4 Continuity Equation

**Fig. 1.**
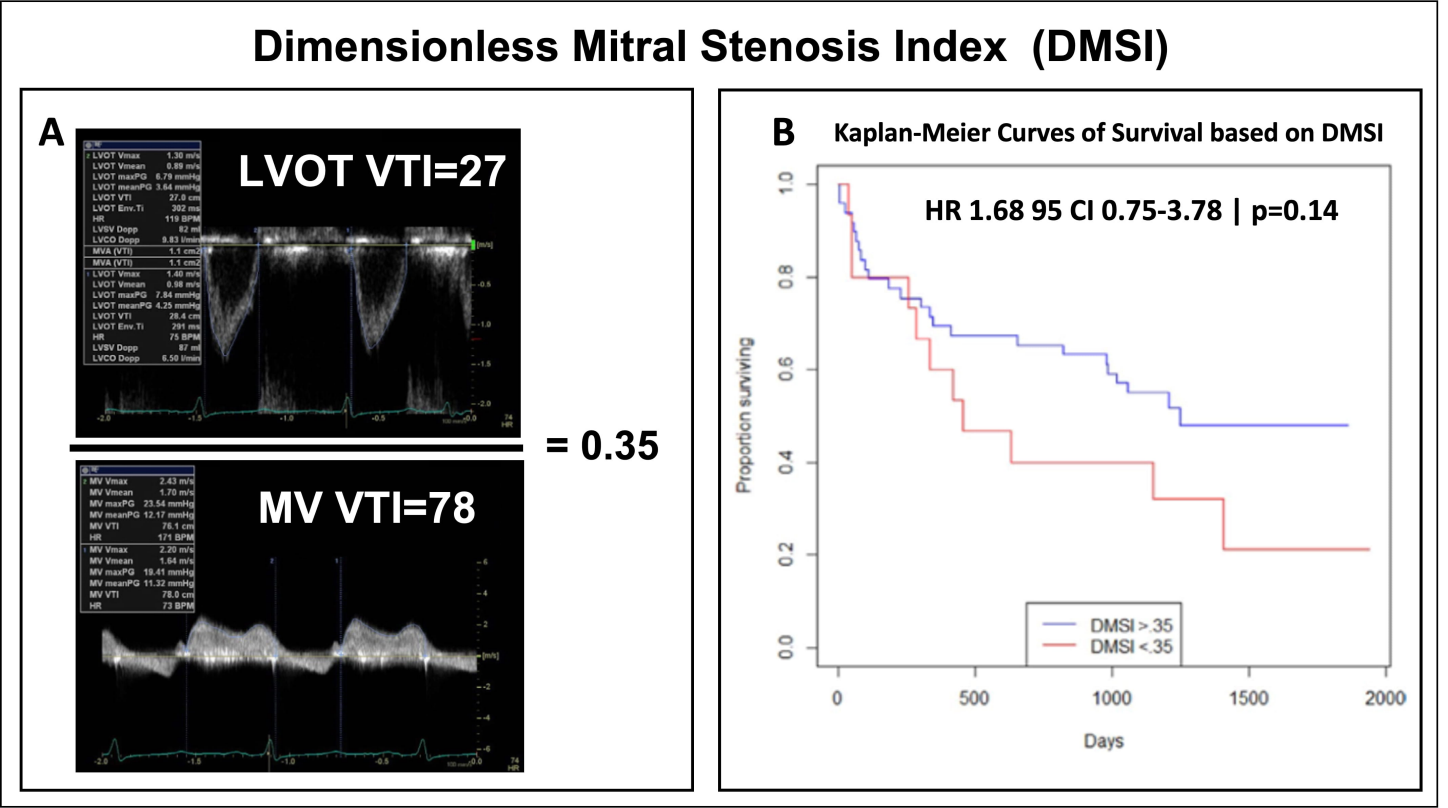
**Dimensionless Mitral Stenosis Index**. (A) Calculation of dimensionless mitral stenosis index (DMSI) which is derived by dividing left ventricle outflow tract (LVOT) velocity time integral (VTI) by MV VTI. (B) Nonsignificant trend toward increased risk of mortality was observed with MVACEQ ≤1.0 ${\mathrm{cm}}^{2}$ and DMSI ≤0.35 with 50% mortality in the DMS cohort at 1 year [25].

The continuity equation is an extrapolation of the concept of conservation of mass, and states that in the absence of valvular regurgitation or intracardiac shunting, the trans-mitral stroke volume (SV) should be equal to the SV determined at the level of the left ventricular outflow tract (LVOT) or right ventricular outflow tract (RVOT) [21]. As SV = valve area x velocity time integral (VTI), one can deduce that MVA = LVOT SV/VTI-MV (in the absence of significant mitral or aortic regurgitation). Calcium may extend into the aorto-mitral curtain affecting calculation of the LVOT cross-sectional area; another source of error when using the continuity equation method is the assumption of a circular shape for the LVOT [24]. To overcome these issues, a dimensionless mitral stenosis index (DMSI) = (LVOT-VTI/MV-VTI) has been proposed (Fig. 1A, [25]). A DMSI of 0.35 to 0.50 is consistent with severe DMS (MVA ≤1.5 ${\mathrm{cm}}^{2}$), and a DMSI <0.35 suggests very severe calcific MS (MVA ≤1.0 ${\mathrm{cm}}^{2}$). A nonsignificant trend toward increased risk mortality was observed with MVACEQ ≤1.0 ${\mathrm{cm}}^{2}$ and DMSI ≤0.35 with 50% mortality in the DMS cohort at 1 year (Fig. 1B) [25]. However, this tool cannot be used in patients with significant left sided regurgitation as VTI across aortic or mitral valve would be higher and this may over or overestimate stenosis severity.

### 2.5 Trans-Mitral Gradient Evaluation (TMG)

Peak and mean trans-mitral gradients are important parameters to assess in DMS. Peak TMG, measured by Continuous Wave Doppler (CW) across the MV using the simplified Bernoulli equation (4 × $v^{2}$), is highly variable and influenced by left atrial compliance and LV diastolic dysfunction [26]. Mean TMG is obtained by tracing the CW envelope of mitral inflow and is more reflective of valvular stenosis. Pulsed wave Doppler at MV leaflet tips is used to measure the early (E) and late (A) diastolic velocities [17]. In one study of patients with severe MAC and a MVA ≤1.5 ${\mathrm{cm}}^{2}$, E velocity was significantly elevated compared to those with MVA >1.5 ${\mathrm{cm}}^{2}$ with no difference in A velocity or E to A ratio between the 2 groups [27].

In patients with RMS, Doppler-derived mean TMG values correlate well with invasive measurements obtained via trans-septal catheterization [28]. Such validation studies are lacking for DMS patients. There is a poor correlation seen between mean gradients and MVA using the continuity equation in DMS [25]. This lack of correlation can be attributed to different issues with trans-mitral flow, increased heart date (i.e., shortened diastole), and decreased left atrial and LV compliance in patients with diastolic dysfunction [17]. Given that the pressure gradient is directly related to the squared function of transvalvular flow rate [29], high cardiac output states in patients with advanced CKD or end-stage renal disease (ESRD) — which are seen in almost 19% of patients with DMS [23], may increase trans-mitral gradients in the absence of significant stenosis [30, 31]. On the other hand, lack of contraction of flow due to tunnel-like morphology as well as low flow state seen in elderly women may lower the trans-mitral gradient in MV and underestimate DMS severity. TMPG ≥8 mmHg in severe DMS (MVA by continuity equation <1.5 ${\mathrm{cm}}^{2}$) was independently associated with 1-year mortality in a study of 200 patients in those with MVA by <1.5 ${\mathrm{cm}}^{2}$ by CEQ, while another study of 5754 patients with mean MV gradient ≥3 mmHg also found an association between MV gradient and mortality (adjusted HR 1.064 per 1 mmHg increase 95% CI 1.049–1.080) [32].

### 2.6 Role of Trans-Esophageal Echocardiography (TEE) and 3-Dimensional Evaluation 

Although transthoracic echocardiography (TTE) can provide a good estimation of the burden of MAC, in severe MAC, the calcification can create an echocardiographic artifact and creates acoustic shadowing but lacks the echo-lucent center which might obscure the valve assessment and [33]. Transesophageal echocardiography (TEE) can provide better visualization of the mitral annulus and left ventricle thickness and can accurately assess the extension of MAC to the surrounding structure [33, 34]. TEE has the superiority of temporal resolution over TTE, and thus it is the method of choice to further evaluate and characterize the MV function, extent of calcification, and the leaflet characterization [35]. Cardiac computed tomography scan on the other hand as higher isotopic spatial resolution with excellent calcification definition [36].

Three-dimensional echocardiography provides multiplanar reconstruction of valvular anatomy and the extension of MAC. However, the presence of high burden calcification might create acoustic shadowing which subsequently hinders the complete analysis of the sub-valvular apparatus or left ventricular outflow tract [35]. To minimize this false measurement, a zoomed view at the tip of the mitral leaflet and using planimetry method to trace the MV orifice when the valve is wide open in the diastolic frame [37]. The other advantages of 3D over 2D include the ability to rotate the images and examine the MV from different perspectives and formulate a relationship between the MV and its surrounding structures [37, 38].

## 3. Effect of Other Valvular Lesions on Assessing Severity of DMS

### 3.1 Effect Tricuspid Valve Flow on Measurement of MS 

It is imperative to evaluate tricuspid valve and pulmonary artery systolic pressure (PASP) as part of the markers of progression of MV disease. In the absence of an alternative explanation of elevated PASP in the setting of MV stenosis, this elevation of PASP could have hemodynamic consequences on the progression of MV stenosis. It is not uncommon to have normal resting PASP even in the presence of severe MV stenosis; however, as the disease progresses, more dilated right ventricle and worsening in the tricuspid regurgitation of tricuspid leaflet tethering could occur [37, 39]. Furthermore, the marked disparity between the valve area between MS and the tricuspid valve being dilated create the paradoxical (leftward) motion of the septum in the diastole given the early diastolic filling across the tricuspid valve [40]. Severe tricuspid regurgitation can reduce right sided cardiac output leading to low-flow, low-gradient pattern makes the accurate assessment of stenosis more difficult [41].

### 3.2 Assessment of DMS Severity in the Setting of Severe Aortic Stenosis

Kato *et al*. [23] showed significant improvement in MVA by CEQ (2.00 ± 0.50 vs. 2.26 ± 0.62 ${\mathrm{cm}}^{2}$*p *< 0.001) in 190 patients with MS (mean gradient >4 mmHg) undergoing aortic valve replacement (AVR) and with a significant decrease in gradient post TAVR (5.2 ± 1.5 vs. 4.7 ± 1.9 mmHg). This is likely partly related to the improvement in LVOT stroke volume but undermines the issue of flow dependency of transmitral gradients and MVA CEQ. However, in patient with true MS, MVA remained <2.0 ${\mathrm{cm}}^{2}$ after AVR, mean TMG and LVOT SV did not change significantly after AVR. In patients with true MS, extension of calcification of the leaflets was more frequently observed and LVOT SV and AVA was smaller than those with pseudo severe stenosis. MVA ≤1.5 ${\mathrm{cm}}^{2}$ CEQ and mitral annular distension ≤1 mm or extension of calcification to both anterior and posterior mitral leaflets suggested the presence of true mitral stenosis. No clear validated diagnostic tool is available to differentiate severe from pseudo severe MS, as those with severe MS have higher mortality post AVR AT 2.9 years and will need treatment for both valves simultaneously or sequentially [42].

Diagnosing significant DMS is crucial as worst outcomes have been reported in patient with severe mitral stenosis undergoing TAVR (higher in-hospital mortality and heart failure-related hospitalization at 1-year in severe MS compared to those without MS [43]) and four-fold increase in cardiovascular death, and a 3.9-fold increase in disabling stroke at 30-days in those with MS [44]. In a study 61 serial echocardiograms in 8 patients with severe DMS undergoing TAVR, persistent elevation of mean TMG (6.7 ± 3 mmHg pr vs. 8.3 ± 1.5 mmHg Post TAVR) and PASP (52 ± 22 vs. 32 ± 8 mmHg, *p* = 0.048) was seen at 2 years when compared to age and sex-matched controls without DMS potentially explaining the increased mortality seen in this group of patients [42].

## 4. Utility of Exercise/Stress Echocardiography of Hemodynamic Measurement

**Fig. 2.**
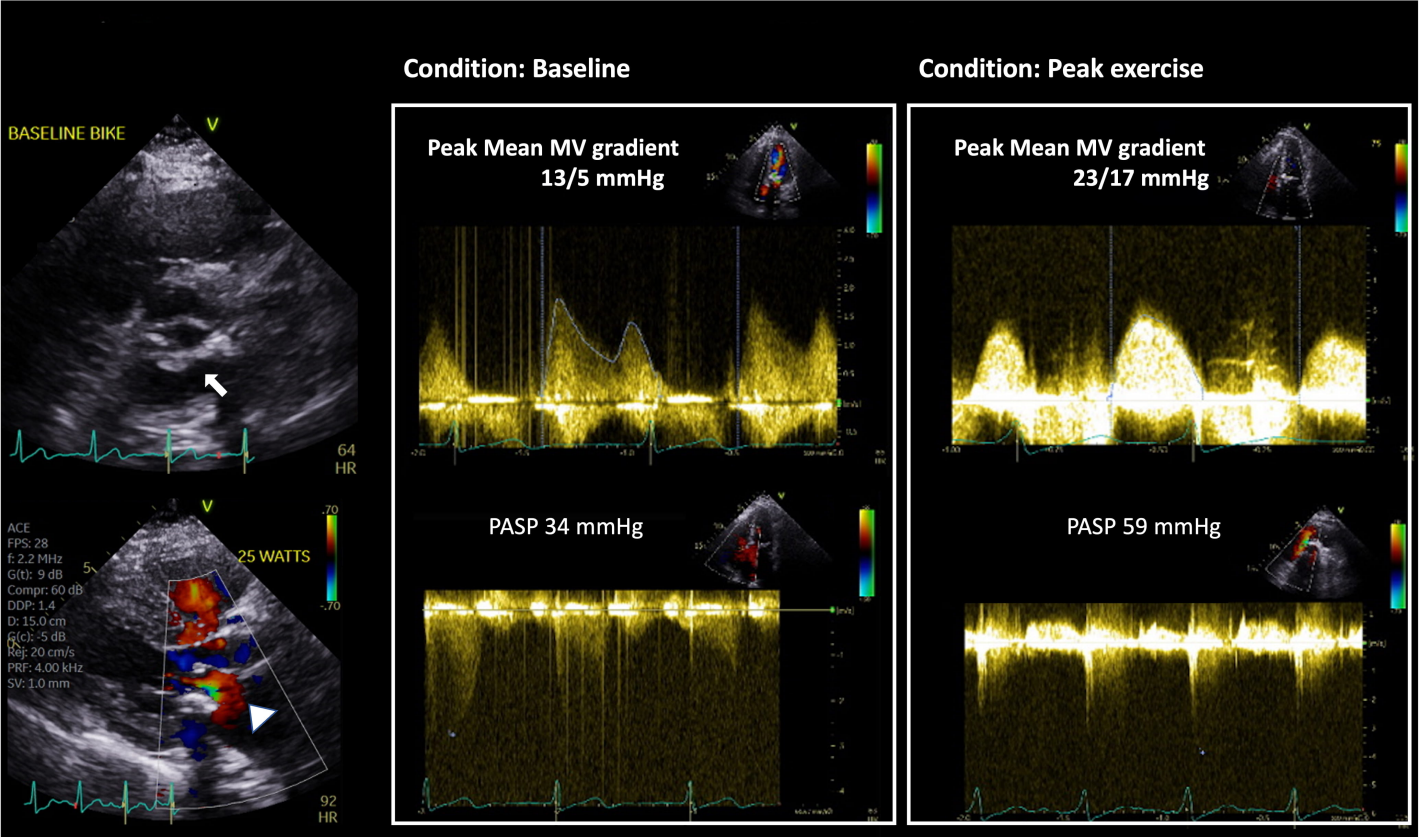
**Utility of Supine Bike Stress Echocardiography in assessment of DMS**. This is an 80-year-old woman with a prior history of coronary artery disease who underwent coronary artery bypass graft surgery and surgical aortic valve replacement with 21 mm Medtronic Mosaic valve 4 years prior. She presented with progressive dyspnea on exertion. Her aortic valve prosthesis showed normal function with pressure gradients unchanged from prior. Of note, severe mitral annular calcification with posterior MV leaflet restriction (arrow) and flow acceleration across the MV in diastole (arrowhead) are seen. MVACEQ is 1.5 ${\mathrm{cm}}^{2}$. Peak/Mean MV gradients are 13/5 mmHg and PASP 34 mmHg at baseline, with increases to 32/17 mmHg and PASP 59 mmHg at 75-Watt supine bicycle stress. CEQ, continuity equation; PASP, Pulmonary artery systolic pressure.

**Fig. 3.**
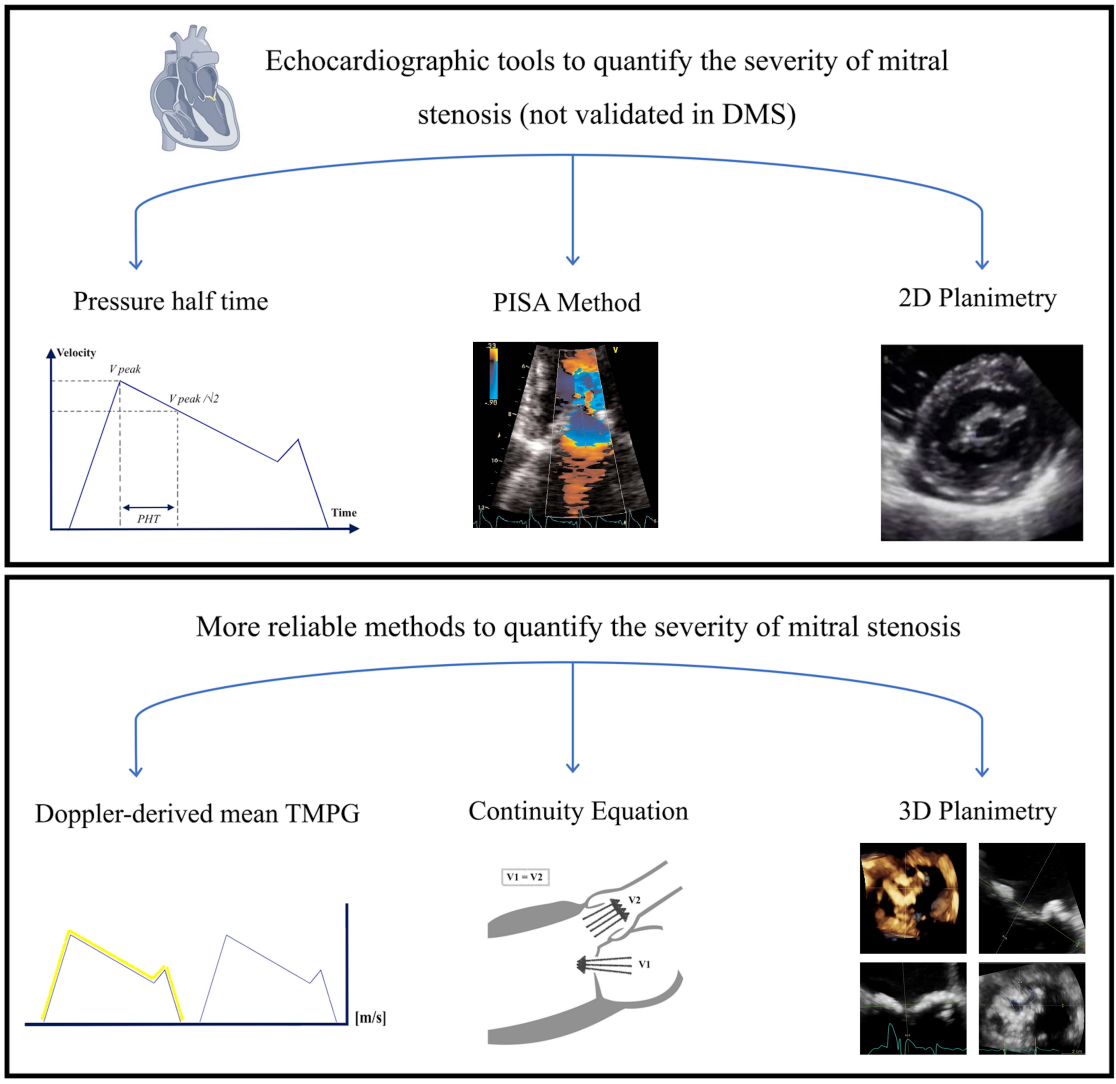
**Echocardiographic tools to quantify the severity of mitral stenosis**. DMS, degenerative mitral stenosis.

In symptomatic patients (NYHA class III or IV) with severe MS (i.e., mitral valve area ≤1.5 ${\mathrm{cm}}^{2}$, Stage D) attribu to extensive MAC, valve intervention may be considered. For patients with symptoms that are disproportionate to the resting MV gradient and/or valve area or in asymptomatic patients, supine bicycle stress echocardiography (BSE) can be useful in assessing the effect of exercise on the behavior of the stenotic valve (Figs. 2 and 3). Exercise increases cardiac output and heart rate and decreases diastolic filling time, and these changes eventually lead to significantly elevated left atrial and pulmonary capillary wedge pressures and an increase in TMG and pulmonary arterial pressure (PAP) [45]. BSE is preferred method over treadmill exercise as BSE provides a more physiological response to stress with a higher elevation of heart rate, valve gradients and PAP [45, 46].

Exercise testing can also bring out symptoms in patients who claim to be asymptomatic. In one study of such patients, in this case with moderate to severe rheumatic disease, BSE produced symptoms in 46% of patients [46]. Another study among patients with DMS, compared 20 patients with severe MAC and restricted valve opening to 20 control subjects matched for age, sex, and LV wall thickness. TMG rose significantly with exercise (17.3 ± 8.4 vs. 5.5 ± 2.5 mmHg at baseline, *p *< 0.0001) and peak PASP exceeded 60 mmHg in 72% of DMS patients versus 26% of controls (*p* = 0.01). The authors concluded that MAC may be an under-recognized cause of dyspnea and exercise intolerance in older patients [47]. If exercise cannot be performed, Dobutamine Stress Echocardiography (DSE) may be useful [48]. In asymptomatic RMS patients PASP elevation above 60 mmHg and rise in TMPG is ≥18 mmHg can help in the decision for intervention [49]. Previously mentioned echocardiographic parameters (i.e., valve area planimetry, PISA and TMG) are utilized to evaluate the MVA.

## 5. Therapeutic Intervention for DMS

Therapeutic intervention for DMS carries significant surgical risk in both MV repair and replacement. There is increased risk of cardiac rupture at an atrioventricular junction or the free wall, and injury to the left circumflex artery in the process of debridement of the MAC [50, 51]. This higher risk is also attributed to the advanced age, higher number of comorbidities, and higher technical difficulty due to excessive calcium on the valve annulus [52]. The complication rate following surgical intervention for DMS is variable. Carpentier *et al*. [51] reported perioperative myocardial infarction in 4.9%, low cardiac output syndrome in 13.1% and other complications such as mediastinal bleeding or high degree atrioventricular block ranged between 3.3%–4.9%. However, in patients with severe MAC and severe mitral valve dysfunction (MS or mitral regurgitation) mitral valve intervention may improve outcomes [53].

Advances in percutaneous valve replacement techniques have led to trials of transcatheter mitral valve replacement (TMVR). TMVR in the presence of severe MAC has been reported to be more challenging due to a higher risk of LV outflow tract obstruction, paravalvular leak, valve thrombosis or valve embolization [54]. In the cumulative experience of TMVR, the highest mortality is seen in patients with MAC [55]. In the Mitral Implantation of TRAnscatheter vaLves (MITRAL) trial, 91 patients were enrolled which all had severe MAC and associated valve dysfunction, and high surgical risk for standard surgical MV replacement. Participants underwent TMVR with a balloon expandable Edwards SAPIEN XT or SAPIEN 3 valve (Edwards Lifesciences, Irvine, California). There was a 1-year mortality rate of 34%. However, survivors had a reduction in New York Heart Association classification to I or II and a drop in TMG to 6 mmHg [56]. Of note, patients with a high risk of LVOT obstruction were excluded. Long-term outcomes of this trial are awaited. Another ongoing trial is the transcatheter mitral valve replacement with the Medtronic Intrepid™ TMVR System in patients with severe symptomatic mitral regurgitation (APOLLO) trial, which involves a MAC cohort [57].

## 6. Conclusions

In the light of emerging percutaneous therapeutic interventions, there is an imminent need for diagnostic tools that provide accurate effective orifice areas in the setting of DMS to assess its severity. In the absence of reliability of MV gradients and PHT that have been traditionally used for RMS, we are limited to the use of the continuity equation and its derivative (i.e., DMSI) along with 3-D MVA for stenosis severity assessment. These tools can also be variable in the setting of low LVOT flow. Effect of low LVOT flow on transmitral gradients and relationship of mean mitral gradients and MVA to flow may help establish new tools that are more accurate for diagnosing DMS. In patients who have symptoms that are out of proportion to the degree of stenosis, a physiological stress test may help determine the physiologic effects of the valve stenosis. New techniques for transcatheter MV replacement appear promising but continue to be associated with high longer-term mortality in patients with MAC.

## Abbreviations

AVR, Aortic valve replacement; BSE, Bicycle stress exercise; CW, Continuous wave; CKD, Chronic kidney disease; ESRD, End-stage renal disease; DMS, Degenerative mitral stenosis; DMSI, Dimensionless mitral stenosis index; DSE, Dobutamine stress echocardiography; DT, Deceleration time; LV, Left ventricle; LVOT, Left ventricular outflow tract; MAC, Mitral annular calcification; MS Mitral Stenosis; MVA, Mitral valve annulus; MV, Mitral valve; PAP, Pulmonary arterial pressure; PASP, Pulmonary arterial systolic pressure; PHT, Pressure half-time; PISA, Proximal iso-velocity surface area; RMS, Rheumatic mitral stenosis; RVOT, Right ventricular outflow tract; SV, Stroke volume; TEE, Trans-esophageal echocardiography; TMVR, Transcatheter mitral valve replacement; TMG, Trans-mitral gradient; TTE, Transthoracic echocardiography; VTI, Velocity time integral.
